# Appendicitis as a presentation of COVID-19: A case report

**DOI:** 10.1016/j.amsu.2021.102719

**Published:** 2021-08-18

**Authors:** Kiran Malbul, Srijana Katwal, Swojay Maharjan, Suraj Shrestha, Roman Dhital, Ashish Prasad Rajbhandari

**Affiliations:** a Nepalese Army Institute of Health Sciences College of Medicine, Sanobharyang, Kathmandu, Nepal; b Maharajgunj Medical Campus, Institute of Medicine, Kathmandu, Nepal; c Nepal National Hospital, Kalanki, Kathmandu, Nepal; d Department of GI and General Surgery, Nepal Medical College Teaching Hospital, Jorpati, Kathmandu, Nepal

**Keywords:** Abdominal pain, Appendicitis, Appendectomy, Case report, COVID-19

## Abstract

**Background:**

Coronavirus disease-19 (COVID-19) is an infectious respiratory disease caused by Severe Acute Respiratory Syndrome Corona Virus 2 (SARS-CoV-2). Respiratory symptoms and flu-like presentation are the most defined clinical manifestations. However, gastrointestinal symptoms with acute abdomen have been reported in a small percentage, occasionally mimicking acute appendicitis. Hence, the diagnosis of COVID-19 should be suspected and investigated in every case of acute abdomen in the present situation.

**Case presentation:**

We report a case of a 25-year-old male who presented with features of acute appendicitis. Despite the equivocal ultrasound results, he was scheduled for an emergency appendectomy for Alvarado's score 7 out of 10, who underwent a successful appendectomy. The patient had initially tested negative on an upper respiratory COVID-19 reverse transcription-polymerase chain reaction (RT-PCR) with normal chest X-ray but few hours after the surgery patient developed a high-grade fever. An RT-PCR for COVID-19 was resent following a suspicion that came out to be positive.

**Clinical discussion:**

Several case reports have suggested a probable association between COVID-19 and appendicitis. This case shows the limited effectiveness of clinical diagnosis for the surgical abdomen in COVID-19 patients as these two conditions share similar symptoms often needing a clinical vigilance.

**Conclusion:**

This case reports acute appendicitis in a patient who tested positive for SARS-CoV-2 subsequently following emergency appendectomy highlighting the acute gastrointestinal presentation of COVID-19. This case exemplifies the necessity to be familiar with the gastrointestinal symptoms of COVID-19 and maintain a high level of suspicion for COVID-19 infection in cases of abdominal pain.

## Highlights

• Acute appendicitis can be one of the presentation of COVID-19.
• Clinicians need to be familiar with the gastrointestinal symptoms of COVID-19.
• Rapid diagnosis of COVID-19 in patients with acute abdominal pain should be done to prevent the virus from spreading.
• A negative RT-PCR for COVID-19 can't totally exclude COVID-19.

## 1 Introduction

Coronavirus disease-19 (COVID-19), a WHO declared a global pandemic, is caused by severe acute respiratory syndrome Coronavirus 2 (SARS-CoV-2) [1]. Fever, dry cough, myalgia, tiredness, dyspnea, and anorexia were common symptoms during the onset of the disease. However, a large number of individuals also reported gastrointestinal symptoms, and anosmia/dysgeusia [2]. Abdominal pain and pathological features resulting in abdominal discomfort in adult Covid-19 infections are reported to be in the region of 2.2–5.8% in cohort studies [2,3]. Few cases of Covid-19 presenting with acute abdomen with features of pancreatitis and appendicitis have also been reported [4,5]. Many studies revealed that fewer or the same number of patients presented with acute appendicitis to the emergency room during the COVID-19 pandemic compared to the non-pandemic period, and those who did, presented with complications [[6], [7], [8]]. We need to consider COVID-19 as a possible diagnosis, even in the event of an abdominal pain syndrome suggesting acute appendicitis. Screening for a co-infection before emergency surgery can modify the treatment and lead to a reassessment of the therapeutic proposal [9]. This case report is in line with the SCARE 2020 criteria [10].

## 2 Case presentation

A 25-year-old male healthcare worker showed himself to the emergency department with acute epigastric pain and an episode of non-projectile, non-bilious vomiting for 6 hours. The pain was sharp, radiating to his right lower abdominal quadrant, aggravated by movement, and unabated by the patient's use of acetaminophen and an antacid. He denied experiencing water brash, pain radiating to the back, diarrhea, fever, burning micturition or respiratory symptoms such as cough, sore throat, and shortness of breath. His past medical, surgical, drug and allergic history was insignificant with no history of similar episodes among family members. He is a nonsmoker who does not drink alcohol or use recreational drugs. On examination, the patient was well-looking, comfortable, and cooperative, with vital signs within normal limits. Per abdominal findings revealed superficial and deep tenderness on the right lower abdominal quadrant with guarding while rebound tenderness, psoas test, and obturator test were negative. Laboratory reports revealed significant leukocytosis (15,010/cumm; reference range 4000–11000/cumm) with neutrophilia (90%; reference range 40–70%) and lymphopenia (08%; reference range 20–45%), increased prothrombin time (20 seconds; reference range 11–16 seconds), raised Alkaline Phosphatase (321U/L; reference range 80–290 U/L) and total protein levels (8.3g/dl; reference range 6–8 g/dl). Nevertheless, amylase, lipase, urine analysis, liver function tests, and renal function tests were unremarkable. His chest x-ray was normal. On abdominal and pelvic ultrasonography (USG), the appendix could not be visualized but echogenic inflamed omentum was seen in the right lower abdominal quadrant.

**Fig. 1:**
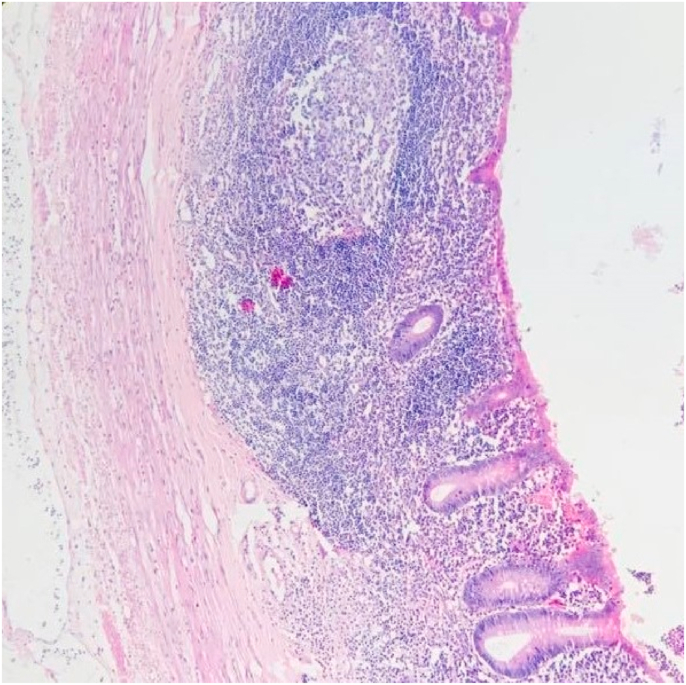
Section shows mucosa lined by columnar epithelium. Lamina propria with mucosal glands. Mucosa and submucosa with lymphoid aggregates. Muscularis layer shows acute inflammatory cells (H&E,X50).

**Fig. 2:**
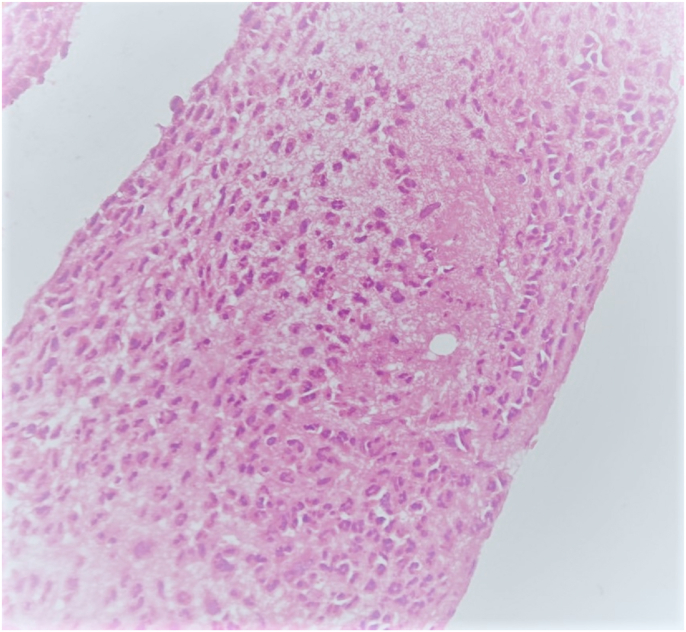
Acute appendicitis: Section shows muscularis showing neutrophilic infiltrate (H&E,x400).

Total Alvarado's score was 7 out of 10 (anorexia-1, nausea-1, right lower abdominal quadrant tenderness-2, leukocytosis-2, the shift of white blood cell count to the left-1) which supported the diagnosis of appendicitis, and opted for an open appendectomy. RT-PCR test for COVID-19 was performed as per the protocol of the hospital for emergency surgery which came out to be negative. Then, under spinal anaesthesia, an open appendectomy was conducted with full COVID-19 precautions. Surgery was performed by general and gastrointestinal surgeon with 7 years of surgical speciality training at private hospital. On gross examination, the appendix was swollen and erythematous containing faecal matter with no signs of perforation. Histopathological examination report revealed acute appendicitis with peri-appendicitis Fig. 1, Fig. 2. Then, intravenous antibiotics ceftriaxone 2 gm IV once daily and metronidazole 500 mg IV three times daily were provided. Six hours after surgery, the patient started to develop cough and fever of 105 °F with chills and rigour and blood pressure of 80/40 mmHg. IV Normal saline and acetaminophen were started with the continuation of IV antibiotics. Repeat blood and urine investigations were sent with the suspicion of systemic infection instead results showed lymphopenia (15%) and thrombocytopenia (92,000/cumm) with increased prothrombin time (14s). The total WBC count was within normal limits. Patient was then planned for abdomen and pelvic USG for possible localized infections and collections. With suspicion of COVID-19 for the cough and fever, the patient's nasopharyngeal and oropharyngeal samples were resent for COVID-19 RT-PCR which later came out to be positive. The patient was then shifted to the COVID-19 isolation ward and was managed as per the COVID-19 management protocol. Ctscan of the chest wasn't performed due to financial constraints but a repeat chest xray didn't show any discrete COVID-19 changes. Due to cost constraints, a CT scan of the chest was not conducted, but a repeat chest x - ray revealed no distinct lesions of COVID-19 indicating a better prognosis.

### 2.1 Final diagnosis and outcome

The final diagnosis of our case was appendicitis with COVID-19 without pneumonia. His clinical conditions progressively improved and were symptomatically relieved after 9 days of isolation and was discharged on 14^th^ day.

### 2.2 Followup

The course after discharge was successful and without complication. There were no traces of fever or respiratory symptoms at the time of discharge. Patient was followed up weekly after discharge for 2 weeks on outpatient visit at hospital.

## 3 Discussion

Gastrointestinal symptoms like nausea, vomiting, abdominal discomfort and diarrhea have been reported in some patients with SARS-COV-2. These GI symptoms varied significantly among different study populations with early or mid-onset along with usual respiratory symptoms [11]. Some studies claim appendicitis to be a presenting symptom apart from the common respiratory symptoms [4,9].

Angiotensin-converting enzyme 2 (ACE2) is an entry point for SARS-COV-2 commonly expressed on the cell membranes of the type II pneumocytes in lungs, ileal and colonic enterocytes in the GI tract, arteries, heart, and kidney cells. It is a key mechanism for receptor-mediated cell entry and replication in SARS-COV-2 [4]. The exact pathophysiology of SARS-COV-2 still needs extensive research; however, the direct virus invasion into enterocytes or indirect virus entrance via the host inflammatory response through the ACE2 receptors explain the wide variety of GI signs and symptoms in COVID-19 [12,13]. In a case report by K. Suwanwongse hypothesized that mucosal injury causing dysregulated inflammatory responses resulting in hypercoagulability and ischemia of enterocytes could explain the possible mechanism of pseudo-appendicitis in COVID-19 [14]. Other studies have shown that viral infections can induce acute appendicitis in a variety of ways, including lymphoid hyperplasia, which leads to appendix blockage, and mucosal ulcerations resulting in subsequent bacterial infection [4].

Multiple case reports had also shown that COVID-19 could present as acute abdominal pain sometimes even mimicking as acute appendicitis with anorexia, nausea, and vomiting [4,11,15,16]. According to U. Saeed's study, 9 out of 76 patients with acute abdominal pain tested positive for COVID-19 [15]. Likewise, two other case reports suggest a probable association between COVID-19 and appendicitis [4,17]. Therefore, this demands a greater vigilance for rapid diagnosis and intervention in individuals with GI symptoms and concomitant SARS-CoV-2 infection.

Similarly, in our case 25-year-old male presented with gastrointestinal symptoms before constitutional and respiratory symptoms and was diagnosed to be COVID-19 positive. Lymphocytopenia and neutrophilia with ‘left shift’ directed our case towards the surgical abdomen but similar conditions were also noticed in SARS-COV-2 infection. Though the findings of Ultrasonography of the abdomen and pelvis were inconclusive, an Alvarado's score of 7 out of 10, focused our case on the acute abdomen. Then, the case underwent a successful open appendectomy with infection prevention and control technique. As per the recent emergency surgery protocol, RTPCR for COVID-19 was done initially which was negative. With the emergence of cough and high fever 6 hours following surgery, several blood investigations were sent for suspicion of systemic infections and ultrasonography of abdomen and pelvis was planned for possible surgical complications. Several other differential diagnosis were considered which included surgically induced inflammation, immune mediated reactions to drugs and medications, as well as other preexising infections. These differentials were soon ruled out as the fever was high grade which started 6 hours post surgery and persisted even with the discontinuation of anesthetic drugs and medications. An RTPCR for COVID-19 was resent following suspicion of COVID-19 for cough and fever, which by surprise came out to be positive. This is in accordance with the Choe (2020) where RTPCR for COVID-19 had been negative initially [18]. Negative RTPCR before surgery could be explained by several factors like inadequate and improper samplings, incubation period, absence of viral particles in the upper respiratory tract in its early stages [[18], [19], [20]].

Our case study shows the limited effectiveness of clinical diagnosis for the surgical abdomen in COVID-19 patients as these two conditions share symptoms such as fever, anorexia, nausea, vomiting, and even acute abdominal pain. It also reflects that SARS-COV-2 could be one of the possible cause of acute abdominal cases such as acute appendicitis. Acute abdomen is often a diagnostic dilemma for physicians. Symptoms and signs are frequently non-specific, and they might conceal life-threatening diseases. Patients may suffer significant repercussions if their pain is misattributed. Although gastrointestinal symptoms are less prevalent in COVID-19, the diagnosis of COVID-19 cannot be ruled out and should be investigated in every case. Furthermore, delaying the treatment of the surgical abdomen can lead to serious complications and increased mortality. In contrast, conducting needless surgery in COVID-19 patients results in iatrogenic morbidity and death, increased pressure on healthcare resources, and increased risk for healthcare personnel participating in operational areas [14]. Therefore, a surveillance system and established principles is necessary for managing suspected COVID-19 cases who require emergency surgery to stop the spread of COVID-19 infection [18].

## 4 Conclusion

In conclusion, during the current SARS- CoV-2 pandemic, clinicians must have a high level of suspicion regarding the possibility of COVID-19 on various clinical manifestations including gastrointestinal symptoms. Methods for rapidly diagnosing COVID-19 in patients with acute abdominal pain should be implemented to take early precautionary steps to prevent the virus from spreading.

## Patient perspective

Our patient grew positively with the decision of surgical approach for the appendicitis. The patient's insight and thoughts regarding COVID-19 and acute appendicitis have improved and is thankful to the whole medical fraternity for helping him to recover.

## Provenance and peer review

Not commissioned, externally peer-reviewed.

## Please state any conflicts of interest

None declared.

## Sources of funding

This study did not receive any kinds of grant or fund.

## Ethical approval

N/A.

## Consent

Written informed consent was obtained from the patient for publication of this case report and accompanying images. A copy of the written consent is available for review by the Editor-in-Chief of this journal on request.

## Author contribution

Please specify the contribution of each author to the paper, e.g. study concept or design, data collection, data analysis or interpretation, writing the paper, others, who have contributed in other ways should be listed as contributors.

Dr. Kiran Malbul and Dr. Srijana Katwal were involved in conception of the study, acquisition of data, drafting and reshaping the initial manuscript, and revising the contents.

Swojay Maharjan, Suraj Shrestha, and Dr. Roman Dhital reshaping the manuscript.

Dr. Ashish Prasad Rajhandari helped in revising the manuscript critically for important intellectual content.

All authors approved the final version of the manuscript and agreed to be accountable for all aspects of the work in ensuring that questions related to the accuracy or integrity of any part of the work are appropriately investigated and resolved.

## Registration of research studies

1. Name of the registry: N/A
2. Unique Identifying number or registration ID: N/A
3. Hyperlink to your specific registration (must be publicly accessible and will be checked): N/A

## Guarantor

Kiran Malbul, Nepalese Army Institute of Health Sciences, Kathmandu −44600, Nepal Email: kiran.malbul04@naihs.edu.np.
